# Hydroxychloroquine levels in patients with systemic lupus erythematosus: whole blood is preferable but serum levels also detect non-adherence

**DOI:** 10.1186/s13075-020-02291-z

**Published:** 2020-09-25

**Authors:** Benoit Blanchet, Moez Jallouli, Marie Allard, Pascale Ghillani-Dalbin, Lionel Galicier, Olivier Aumaître, François Chasset, Véronique Le Guern, Frédéric Lioté, Amar Smail, Nicolas Limal, Laurent Perard, Hélène Desmurs-Clavel, Du Le Thi Huong, Bouchra Asli, Jean-Emmanuel Kahn, Laurent Sailler, Félix Ackermann, Thomas Papo, Karim Sacré, Olivier Fain, Jérôme Stirnemann, Patrice Cacoub, Gaelle Leroux, Judith Cohen-Bittan, Jérémie Sellam, Xavier Mariette, Claire Goulvestre, Jean Sébastien Hulot, Zahir Amoura, Michel Vidal, Jean-Charles Piette, on behalf of the PLUS Group, Leonardo Astudillo, Cristina Belizna, Nadia Belmatoug, Olivier Benveniste, Audrey Benyamine, Holly Bezanahary, Patrick Blanco, Bahram Bodaghi, Pierre Bourgeois, Benoît Brihaye, Emmanuel Chatelus, Richard Damade, Eric Daugas, Christian De Gennes, Jean-François Delfraissy, Céline Delluc, Aurélien Delluc, Pierre Duhaut, Alain Dupuy, Isabelle Durieu, Hang Korng Ea, Dominique Farge, Christian Funck-Brentano, Frédérique Gandjbakhch, Justine Gellen-Dautremer, Bertrand Godeau, Cécile Goujard, Catherine Grandpeix, Claire Grange, Lamiae Grimaldi, Gaëlle Guettrot-Imbert, Loïc Guillevin, Eric Hachulla, Jean-Robert Harle, Julien Haroche, Pierre Hausfater, Jean Jouquan, Gilles Kaplanski, Homa Keshtmand, Mehdi Khellaf, Olivier Lambotte, David Launay, Philippe Lechat, Hervé Levesque, Olivier Lidove, Eric Liozon, Kim Ly, Matthieu Mahevas, Kubéraka Mariampillai, Alexis Mathian, Karin Mazodier, Marc Michel, Nathalie Morel, Luc Mouthon, Lucile Musset, Rokiya Ngack, Jacques Ninet, Eric Oksenhendler, Jean-Luc Pellegrin, Olivier Peyr, Anne-Marie Piette, Vincent Poindron, Jacques Pourrat, Fabienne Roux, David Saadoun, Sabrinel Sahali, Bernadette Saint-Marcoux, Françoise Sarrot-Reynauld, Yoland Schoindre, Damien Sene, Jacques Serratrice, Pascal Seve, Jean Sibilia, Claude Simon, Christelle Sordet, Benjamin Terrier, Salim Trad, Jean-François Viallard, Elisabeth Vidal, Bertrand Wechsler, Pierre-Jean Weiller, Noémie Jourde-Chiche, Nathalie Costedoat-Chalumeau

**Affiliations:** 1 AP-HP, Hôpital Cochin, Biologie du médicament – Toxicologie, 27 rue du Faubourg Saint-Jacques, 75014 Paris, France; 2 UMR8038 CNRS, U1268 INSERM, Faculty of Pharmacy, University Paris Descartes, PRES Sorbonne Paris Cité, Paris, France; 3 Service de Médecine interne, Hôpital Hédi Chaker, Sfax, Tunisie; 4 Université Paris-Diderot, Sorbonne Paris-Cité, F-75205 Paris, France; 5 AP-HP, Hôpital Bichat Claude-Bernard, service de médecine interne, 46 rue Henri-Huchard, 75018 Paris, France; 6 AP-HP, Hôpital Pitié-Salpêtrière, Département d’immunologie, 47-83 Boulevard de l’Hôpital, 75651 Paris Cedex 13, France; 7 Université Paris Diderot, Sorbonne Paris Cité, Paris, France; 8 AP-HP, Hôpital Saint Louis, service d’immunologie clinique, 1 avenue Claude Vellefaux, 75010 Paris, France; 9 Université de Clermont-Ferrand, 63003 Clermont-Ferrand, France; 10 CHU Clermont-Ferrand, Hôpital Gabriel Montpied, service de médecine interne, 58 rue Montalembert, 63003 Clermont-Ferrand cedex1, France; 11 UPMC, Université Paris 6, Paris, France; 12 AP-HP, Hôpital Tenon, service de dermatologie allergologie, 4 rue de la Chine, 75020 Paris, France; 13 AP-HP, Hôpital Cochin, Centre de référence maladies auto-immunes et systémiques rares, service de médecine interne, 27 rue du Faubourg Saint-Jacques, 75014 Paris, France; 14 Université de Paris, F-75205 Paris, France; 15 AP-HP, Hôpital Lariboisière, service de rhumatologie, DMU Locomotion, 2 rue Ambroise Paré, 75010 Paris, France; 16 CHU Amiens, Hôpital Nord, service de médecine interne, Place Victor Pauchet, 80000 Amiens, France; 17 AP-HP, Hôpital Henri Mondor, service de médecine interne, 51 avenue du Maréchal de Tassigny, 94000 Créteil, France; 18 Centre Hospitalier Saint Joseph Saint Luc, service de médecine interne, 20 quai Claude Bernard, 69007 Lyon, France; 19 Hospices Civils de Lyon, Groupement Hospitalier Edouard Herriot, service de médecine interne, 5 place d’Arsonval, 69003 Lyon, France; 20 AP-HP, Hôpital Pitié-Salpêtrière, Centre de référence pour le Lupus Systémique et le syndrome des Antiphospholipides, service de médecine interne, 47-83 Boulevard de l’Hôpital, 75651 Paris Cedex 13, France; 21 Servie de Médecine Interne, Hôpital Ambroise Paré, Université Paris Saclay, 9 Avenue Charles de Gaulle, 92104 Boulogne-Billancourt, France; 22 Université Paul-Sabatier, Toulouse, France; 23 CHU Toulouse, Hôpital Purpan, Service de Médecine Interne, Place Dr Baylac, F-31059 Toulouse, France; 24 Hôpital Foch, Service de médecine interne, 92150 Suresnes, France; 25 Sorbonne Université, Hôpital Saint Antoine, APHP, service de médecine interne, F 75012 Paris, France; 26 Hôpitaux Universitaires de Genève, Service de Médecine interne Générale, Avenue Gabrielle Perret Gentil 4, CH-1211 Geneva, Switzerland; 27 AP-HP, Hôpital Pitié-Salpêtrière, Centre de référence maladies auto-immunes et systémiques rares, service de médecine interne 2, 47-83 Boulevard de l’Hôpital, 75651 Paris Cedex 13, France; 28 AP-HP, Hôpital Pitié-Salpêtrière, service de gériatrie, 47-83 Boulevard de l’Hôpital, 75651 Paris Cedex 13, France; 29 AP-HP, Hôpital Saint Antoine, Service de Rhumatologie, 184 Rue du Faubourg Saint-Antoine, 75012 Paris, France; 30 Service de Rhumatologie, Hôpitaux Universitaires Paris-Sud, AP-HP, Université Paris-Sud, INSERM UMR 1184, Paris, France; 31 AP-HP, Hôpital Cochin, service d’immunologie biologique, 27 rue du Faubourg Saint-Jacques, 75014 Paris, France; 32 INSERM, UMRS 970, Paris Cardiovascular Research Center, Paris, France; 33 Aix-Marseille Univ, C2VN, INSERM 1263, INRA 1260 ; AP-HM, Centre de Néphrologie et Transplantation Rénale, Marseille, France; 34 Université Paris-Descartes, Paris, France; 35 INSERM U 1153, Center for Epidemiology and Statistics Sorbonne Paris Cité (CRESS), Paris, France

**Keywords:** Hydroxychloroquine, Systemic lupus erythematosus, Serum, Drug monitoring, Adherence

## Abstract

**Background:**

Hydroxychloroquine (HCQ) levels can be measured in both serum and whole blood. No cut-off point for non-adherence has been established in serum nor have these methods ever been compared. The aims of this study were to compare these two approaches and determine if serum HCQ cut-off points can be established to identify non-adherent patients.

**Methods:**

HCQ levels were measured in serum and whole blood from 573 patients with systemic lupus erythematosus (SLE). The risk factors for active SLE (SLEDAI score > 4) were identified by multiple logistic regression. Serum HCQ levels were measured in 68 additional patients known to be non-adherent, i.e. with whole-blood HCQ < 200 ng/mL.

**Results:**

The mean (± SD) HCQ levels were 469 ± 223 ng/mL in serum and 916 ± 449 ng/mL in whole blood. The mean ratio of serum/whole-blood HCQ levels was 0.53 ± 0.15. In the multivariate analysis, low whole-blood HCQ levels (*P* = 0.023), but not serum HCQ levels, were independently associated with active SLE.

From the mean serum/whole-blood level ratio, a serum HCQ level of 106 ng/mL was extrapolated as the corresponding cut-off to identify non-adherent patients with a sensitivity of 0.87 (95% CI 0.76–0.94) and specificity of 0.89 (95% CI 0.72–0.98).

All serum HCQ levels of patients with whole-blood HCQ below the detectable level (< 20 ng/mL) were also undetectable (< 20 ng/mL).

**Conclusions:**

These data suggest that whole blood is better than serum for assessing the pharmacokinetic/pharmacodynamic relation of HCQ. Our results support the use of serum HCQ levels to assess non-adherence when whole blood is unavailable.

## Key points

- The mean ratio of serum/whole-blood levels of HCQ was 0.53 ± 0.15.
- Whole blood appears to be better than serum for assessing the PK/PD relation of HCQ.
- Serum HCQ levels can be also used to assess non-adherence.

## Introduction

Hydroxychloroquine (HCQ) is widely used in systemic lupus erythematosus (SLE) because of its efficacy in preventing SLE flares, diabetes mellitus, thrombotic events, dyslipidaemia, and overall damage accrual in SLE patients [1, 2]. It may therefore improve survival in SLE [3].

Over the past decade, the ease of whole-blood HCQ assays in hospital laboratories has contributed to the increased use of whole-blood HCQ monitoring in daily clinical practice. Studies have reported relations between the whole-blood HCQ level and clinical outcomes, including but not limited to flare onset and gastrointestinal side effects [4–11]. Although the large French multicentre randomised prospective PLUS study failed to demonstrate the benefit of whole-blood HCQ monitoring for adjustment of daily HCQ dosage [8], it confirmed the pharmacokinetic/pharmacodynamic (PK/PD) relation. Even if most PK/PD studies have been performed in whole blood, others have reported interesting results in serum [12] and thus created some uncertainty about which biological matrix is most suitable for monitoring HCQ levels in the bloodstream. As far as we know, no study has compared the interest of serum and whole-blood HCQ levels in this setting.

Perhaps more importantly, several studies have shown the interest of measuring HCQ levels for identifying non-adherent patients [5, 6, 11, 12–18]. In the first study published in 2007, we retrospectively validated the cut-off of HCQ < 200 ng/mL in whole blood to identify severe non-adherence to treatment [15]. Since then, others have proposed thresholds of 500 ng/mL [4], 100 ng/mL [16, 17, 19], 15 ng/mL [11], and undetectable whole-blood HCQ levels, while others have used our cut-off of 200 ng/mL [6]. Still, others have used serum levels with cut-offs of 100 ng/mL [18] or < 15 ng/mL to define non-adherence (or even 15 to 500 for suboptimal adherence). [12] Apart from our first study, no cut-off points have been validated in patients, and serum and whole-blood levels have not been compared. Because of the strong interest in retrospectively assessing severe non-adherence in both clinical trials and large cohort of patients, and as frozen serum samples are more widely available than frozen whole-blood samples, a cut-off point to identify non-adherence in serum would be very welcome.

Finally, HCQ shows wide interindividual variability in its pharmacokinetics. Different factors, such as body mass index (BMI), are known to contribute to this variability [20], and identifying the optimal dose remains a challenge. The starting daily dose of HCQ is usually based on total body weight (TBW). Given the increasing worldwide prevalence of obesity [21], it is essential to identify the best size descriptor to calculate the most appropriate starting dose of HCQ. Alternate weight descriptors, such as ideal body weight (IBW, based on height, gender, and age) and lean body mass (LBM, calculated by subtracting body fat weight from total body weight), are used for some drugs with weight-base dosing to prevent drug overexposure [22]. Additionally, in the past, the use of IBW for HCQ dosing has been suggested to prevent retinopathy [23, 24]. No data are available regarding the respective relations of TBW, LBM, and IBW to HCQ levels.

The aims of this study were (a) to compare the suitability of serum with that of whole blood for monitoring HCQ, (b) to assess whether a serum HCQ cut-off can be determined to identify severely non-adherent patients, and (c) to investigate the relation between whole-blood HCQ in SLE patients and different weight descriptors, such as TBW, LBM, and IBW.

## Methods

### Patients

**Fig. 1:**
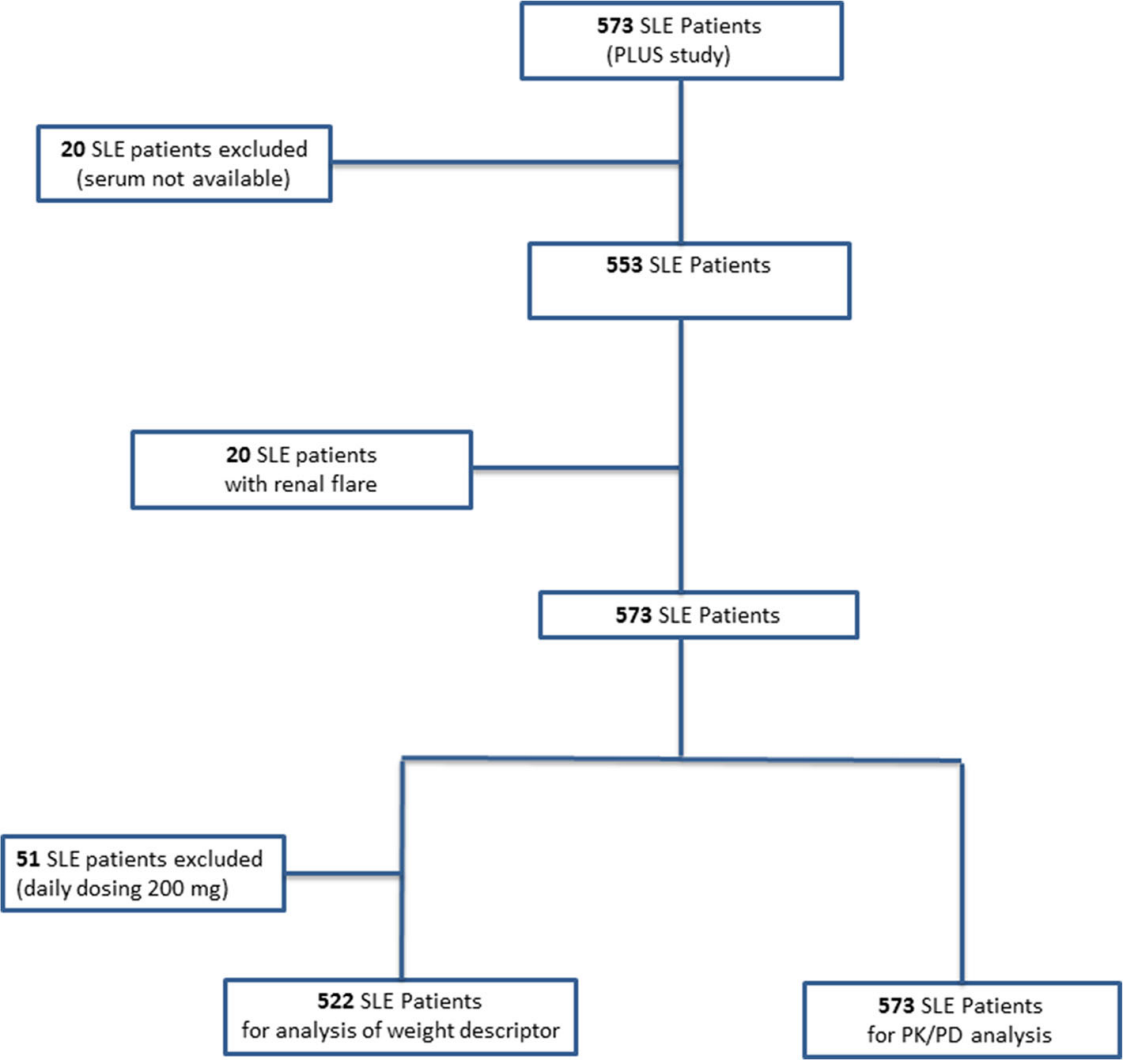
Study flow chart

All patients had SLE according to the American College of Rheumatology (ACR) Classification Criteria [25] and all had been prescribed HCQ (200 or 400 mg/day) for at least 6 months, without dose modification for 2 months. Three sets of patient data were used. First, we used available serum (*n* = 553) from the PLUS Study (Plaquenil LUpus Systemic: PLUS study, ClinicalTrials.gov number, NCT0041336) [9], a French randomised, double-blinded, placebo-controlled, multicentre trial that evaluated the interest of adapting the daily HCQ dose to blood HCQ levels in 573 SLE patients. Known non-adherence to HCQ treatment was an exclusion criterion in the PLUS study, as were severe flares. Accordingly, we used serum and whole blood from 20 additional patients included in a biobank of SLE patients with renal flares (DC-2012-1704, Laboratory of Immunology and Department of Nephrology, Hôpital de la Conception, AP-HM, Marseille). Third, since the exclusion of known non-adherent patients from the PLUS study meant that it included few patients with whole-blood HCQ levels < 200 ng/mL (*n* = 34), we analysed serum (remaining in the immunology laboratory) from 34 non-adherent patients (whole-blood HCQ levels < 200 ng/mL) followed in daily clinical practice at Cochin hospital. Figure 1 presents the study flow chart.

### Ethic statement

The PLUS Study was in compliance with the Declaration of Helsinki and approved by the local medical ethical board. All patients included in the PLUS Study or in the biobank (DC-2012-1704) of SLE patients had provided written informed consent. According to French regulations, written informed consent was not required for blood samples collected in the 33 patients followed in daily clinical practice.

### Data

Laboratory data including haematological (leucocytes, neutrophils, lymphocytes, platelets, and haemoglobin) and immune (plasma protein levels of complement components C4 and C3, anti-DNA antibodies) parameters were recorded. Creatinine clearance was estimated with the Cockroft-Gault formula. IBW was calculated as previously reported [26], and LBM was expressed in kg according to Janmahasatian’s equation [27]: LBM_female_ = (9270 × TBW)/(8780 + (244 × BMI)); LBM_male_ = (9270 × TBW)/(6680 + (216 × BMI)).

### Drug assay

Serum drug levels were always analysed from samples collected simultaneously with those for whole-blood HCQ and DCQ (desethylchloroquine) measurement. After centrifugation (4000 rpm, 5 min), serum was collected, and then stored at − 20 °C until analysis. All determinations of HCQ and DCQ levels in serum were performed in the laboratory of Cochin Hospital. The method used was adapted from a previously published method [28]. The intraday and interday precision of HCQ and DCQ assays in serum ranged from 4.3 to 10.3%. The lower limit of quantification in serum was 20 ng/mL for both HCQ and DCQ.

Measurements of whole-blood HCQ and DCQ levels were already available from the PLUS study (pharmacological laboratory, Centre Hospitalier Universitaire Pitié-Salpêtrière). For the additional patients, whole-blood HCQ and DCQ levels were assayed at Cochin Hospital. Both laboratories use two analytical methods with demonstrated interchangeability [28]. The lower limit of quantification in whole blood was 20 ng/mL for both HCQ and DCQ. Finally, the composite level was defined as the sum of the HCQ and DCQ levels.

### Statistical analysis

For descriptive statistics, qualitative variables were expressed as numbers with percentages and quantitative variables as means ± their standard deviations. Correlations between HCQ, DCQ, and composite levels in serum and whole blood were assessed with Spearman’s correlation coefficient. The univariate analysis of risk factors for active SLE (defined as SELENA-SLEDAI score > 4) used the two-sample Wilcoxon test for quantitative variables and the chi-square test for qualitative variables. The following variables were tested: sex, age, active smoking, treatment by corticosteroids and by immunosuppressants, drug levels (HCQ, DCQ) in both serum and whole blood, BMI, haemoglobin, platelets, leucocytes, lymphocytes, and neutrophils. Variables with *P* values < 0.10 were entered into a multivariate stepwise logistic regression analysis, and the final model included the variables with Wald test *P* values < 0.05. All tests were two-tailed, with *P* significant at < 0.05, and 95% confidence intervals (95% CI) are reported where appropriate. All computations were performed with software SPSS 17 (IBM, France).

## Results

### Pharmacokinetic data

**Fig. 2:**
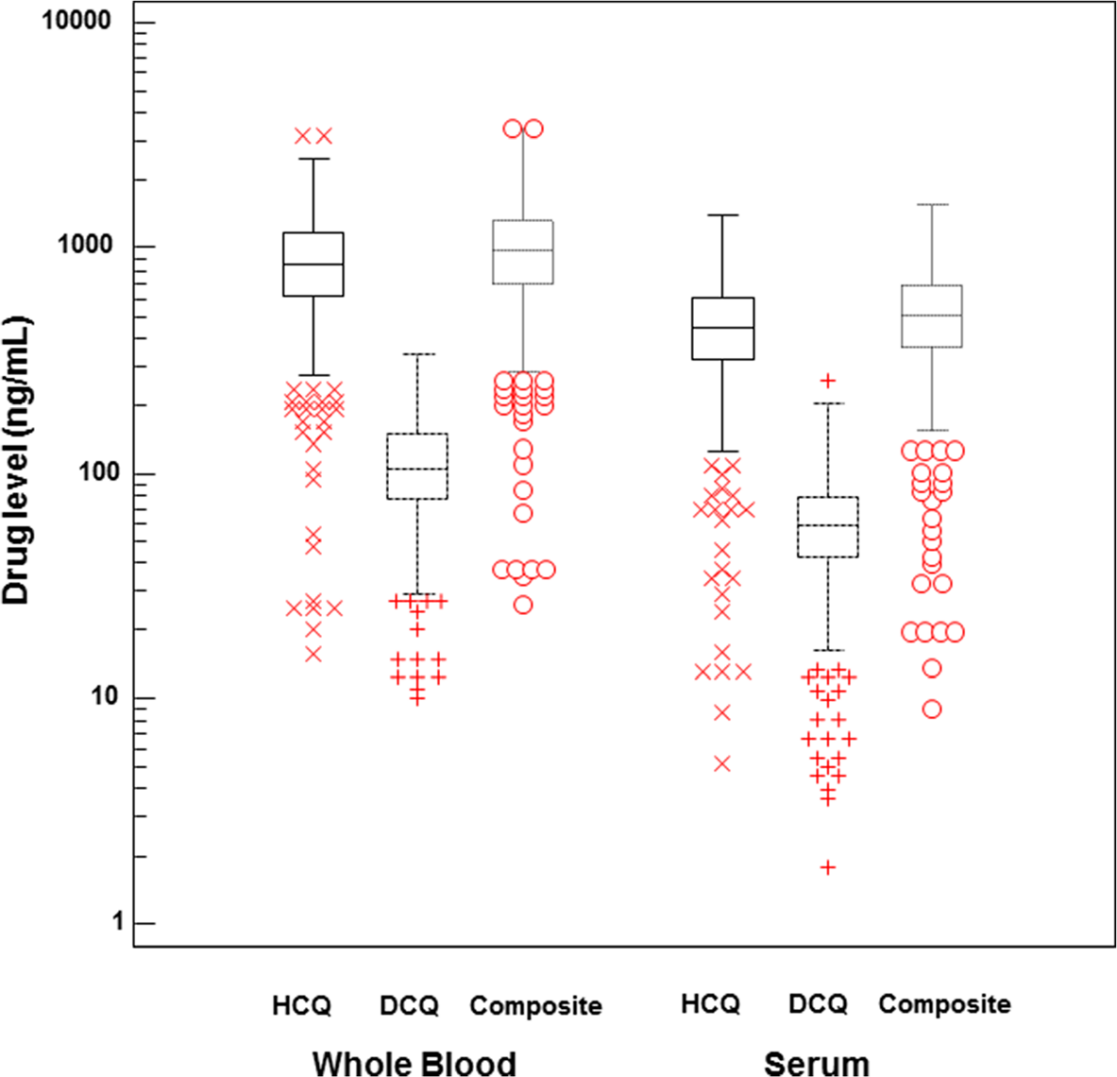
Serum and whole-blood levels of hydroxychloroquine (HCQ), desethylchloroquine (DCQ), and composite (HCQ+DCQ) in log scale

**Fig. 3:**
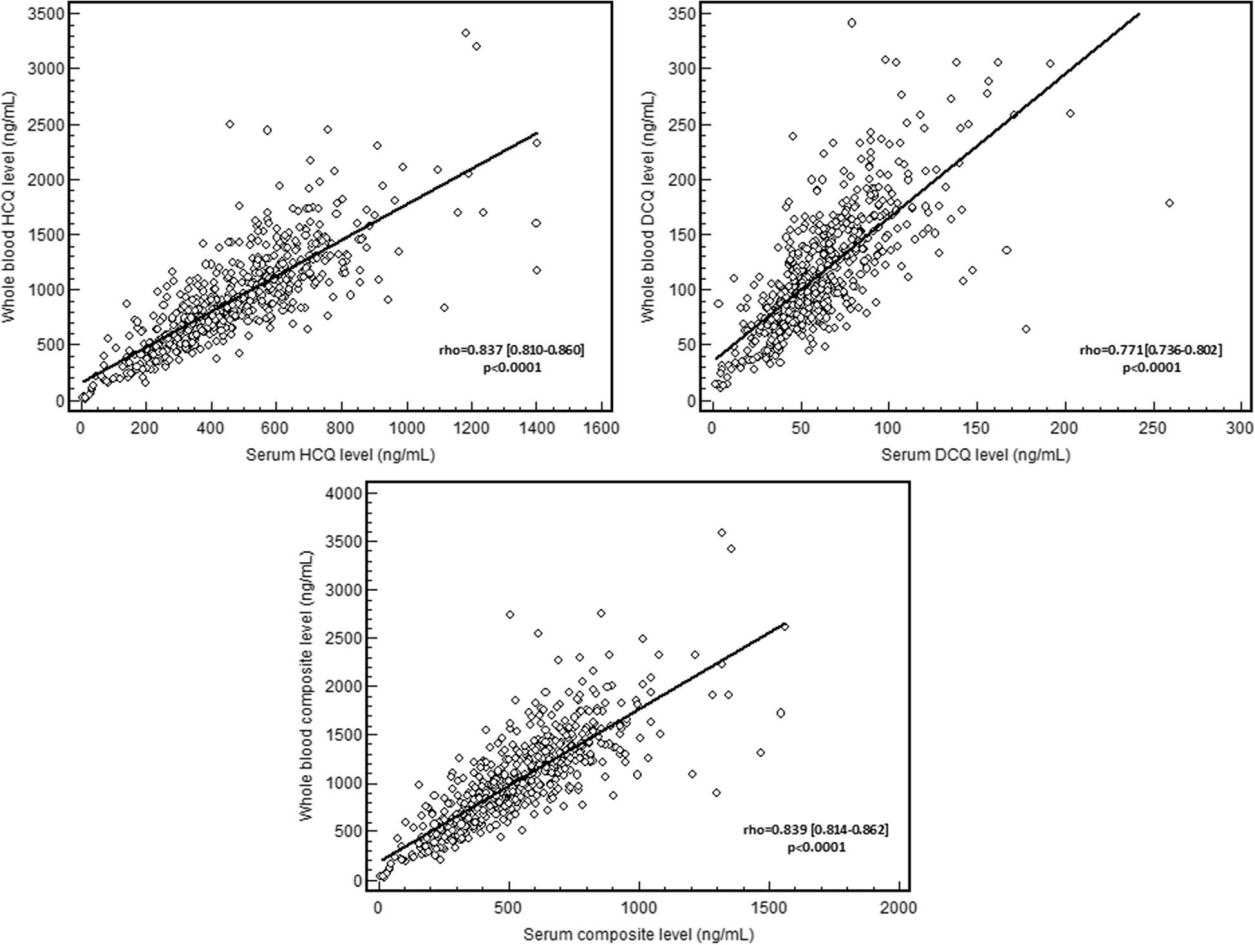
Correlation between serum and whole-blood levels of hydroxychloroquine (HCQ), desethylchloroquine (DCQ), and composite (HCQ+DCQ)

The PK/PD study analysed data from 553 patients included in the PLUS study with available serum HCQ measurements and the 20 patients with renal flares, for a total of 573 SLE patients (Table 1). The HCQ, DCQ, and composite levels in serum were respectively 469 ± 223 ng/mL (CV = 47.6%), 63 ± 31 ng/mL (CV = 50.2%), and 532 ± 249 ng/mL (CV = 46.8%), and in whole blood 916 ± 449 ng/mL (coefficient of variation, CV = 49.1%), 116 ± 55 ng/mL (CV = 48.0%), and 1032 ± 493 ng/mL (CV = 47.8%) (Fig. 2). The mean ratio of serum to whole-blood levels for HCQ and DCQ were 0.53 ± 0.15 (CV = 28.9%) and 0.57 ± 0.21 (CV = 37.0%), respectively. A strong positive correlation was found between serum to whole-blood levels of HCQ (rho = 0.837 [95% CI 0.810–0.860], *P* < 0.0001), of DCQ (rho = 0.771 [95% CI 0.736–0.802], *P* < 0.0001), and to the composite level of both (rho = 0.839 95% CI 0.814–0.862], *P* < 0.0001; Fig. 3).

**Table 1:** Clinical and demographic characteristics of the analysis cohort (*n* = 573)

Covariables	
**Age at diagnosis (years)**	29.3 ± 11.9
**Female sex,** ***n*** **(%)**	520 (91.3)
**Geographical origin,** ***n*** **(%)**	
Europe	335 (58.5)
Sub-Saharan Africa and West Indies (Antilles)	99 (17.3)
North Africa	82 (14.3)
Asia	49 (8.6)
Other	8 (1.4)
Total body weight (kg)	64.9 ± 14.1
BMI (kg/m^2^)	24.0 ± 4.8
Lean body mass (kg)	41.5 ± 7.9
Active smoking, *n* (%)	130 (22.7)
Immunosuppressants, *n* (%)	
Corticosteroids	373 (65.1)
Other^a^	103 (18.0)
SLEDAI score	2.4 ± 3.2
**Clinical manifestations**	
Photosensitivity	328 (57.2)
Malar rash	276 (48.2)
Discoid lupus	64 (11.2)
Arthritis	506 (88.3)
Oral ulcers	96 (16.8)
Haematological manifestations	354 (61.8)
Serositis	146 (25.5)
Nephropathy	176 (30.7)
Neuropsychiatric manifestations	37 (6.5)
HCQ daily dosing, *n* (%)	
400 mg/day	522 (91.1)
200 mg/day	51 (8.9)
**Biological characteristics**	
Leukocytes (× 10^9^/l)	6.3 ± 2.4
Neutrophils (× 10^9^/l)	4.4 ± 2.2
Lymphocytes (× 10^9^/l)	1. 5 ± 0.7
Platelets (× 10^9^/l)	253 ± 75
Haemoglobin (g/dL)	13.1 ± 1.4
Creatinine clearance (mL/min)	103 ± 32
Mild renal dysfunction^†^, *n* (%)	224 (39.1)
Moderate renal dysfunction^‡^, *n* (%)	17 (3)
Plasma C3 level (g/L)	1.00 ± 0.23
Plasma C4 level (g/L)	0.019 ± 0.08

*BMI* body mass index, *HCQ* hydroxychloroquine, *SLEDAI* SLE Disease Activity Index

Quantitative variables are expressed as mean ± standard deviation

^a^Other include azathioprine, cyclophosphamide, methotrexate, and mycophenolate mofetil

^†^Creatinine clearance between 60 and 90 mL/min

^‡^Creatinine clearance between 30 and 60 mL/min

### PK/PD relation

In the univariate analysis (Table 2), the SLEDAI score > 4 was associated with treatment by corticosteroids (*P* = 0.001) and by immunosuppressants (*P* = 0.003), as well as the serum HCQ level (*P* = 0.008), the whole-blood HCQ level (*P* = 0.001), haemoglobin level (*P* < 0.001), and leucocyte count (*P* = 0.036). In the multivariate analysis, treatment by corticosteroids (*P* = 0.044) and by immunosuppressants (*P* = 0.027) as well as low whole-blood HCQ levels (*P* = 0.023) and haemoglobin (*P* = 0.009) were identified as independently associated with active SLE, but the association with serum HCQ levels disappeared.

**Table 2:** Risk factors associated with active systemic lupus erythematosus (*n* = 573)

	Univariate analysis			Multivariate analysis		
	SLEDAI ≤ 4 (*n* = 492)	SLEDAI > 4 (*n* = 81)	*P* value	Odds ratio	CI95%	*P* value
Male sex, *n* (%)	41 (8.3)	9 (11.1)	0.4			
Age (years)	29.1 ± 12	29.9 ± 10	0.58			
Active smoking, *n* (%)	108 (22)	22 (27.2)	0.3			
Corticosteroids, *n* (%)*	307 (62.4)	66 (81.5)	**0.001**	2.033	1.019–4.056	0.044
Immunosuppressants, *n* (%)*	79 (16.1)	24 (29.6)	**0.003**	1.999	1.081–3.697	0.027
HCQ whole-blood level (ng/mL)	940.8 ± 448	765.9 ± 426	**0.001**	0.999	0.997–1.000	0.023
HCQ serum level (ng/mL)	479.9 ± 218	404.9 ± 244	**0.008**			
DCQ whole-blood level (ng/mL)	116.7 ± 54	108.8 ± 59	0.2			
DCQ serum level (ng/mL)	63.6 ± 31	56.3 ± 33	0.051			
BMI (kg/m^2^)	23.9 ± 4.7	24.5 ± 5.3	0.64			
Haemoglobin (g/dL)	13.2 ± 1.3	12.45 ± 1.4	**< 0.001**	0.768	0.630–0.937	0.009
Platelets (cells/mm^3^)	252.5 ± 72.5	254.5 ± 91	0.61			
Leukocytes (cells/mm^3^)	6400 ± 2380	5798 ± 2588	**0.036**			
Lymphocytes (cells/mm^3^)	1466 ± 705	1348 ± 726	0.13			
Neutrophils (cells/mm^3^)	4412 ± 2195	3966 ± 2488	0.069			

*BMI* body mass index, *CI95%* confidence interval 95%, *DCQ* desethylchloroquine, *HCQ* hydroxychloroquine

*Multi-colinearity between corticosteroids and immunosuppressants was assessed. The VIF coefficient (= 1.071) excludes any colinearity between these two variables

### Comparison of serum and whole-blood HCQ levels in non-adherent patients

Given that the mean ratio of serum/whole-blood HCQ levels was 0.53 in our PK/PD cohort, we calculated by extrapolation that serum HCQ cut-offs of 106 and 53 ng/mL would correspond to 200 and 100 ng/mL of HCQ in whole blood, respectively. After adding 34 patients with whole-blood HCQ levels below 200 ng/mL, we had a total of 68 serum samples from patients with severe non-adherence defined by whole-blood HCQ levels < 200 ng/mL. To explore false positives with a serum HCQ cut-off of 106 ng/mL, we used the whole-blood samples with values between 200 and 300 ng/mL (*n* = 25) from our PK/PD cohort.

**Fig. 4:**
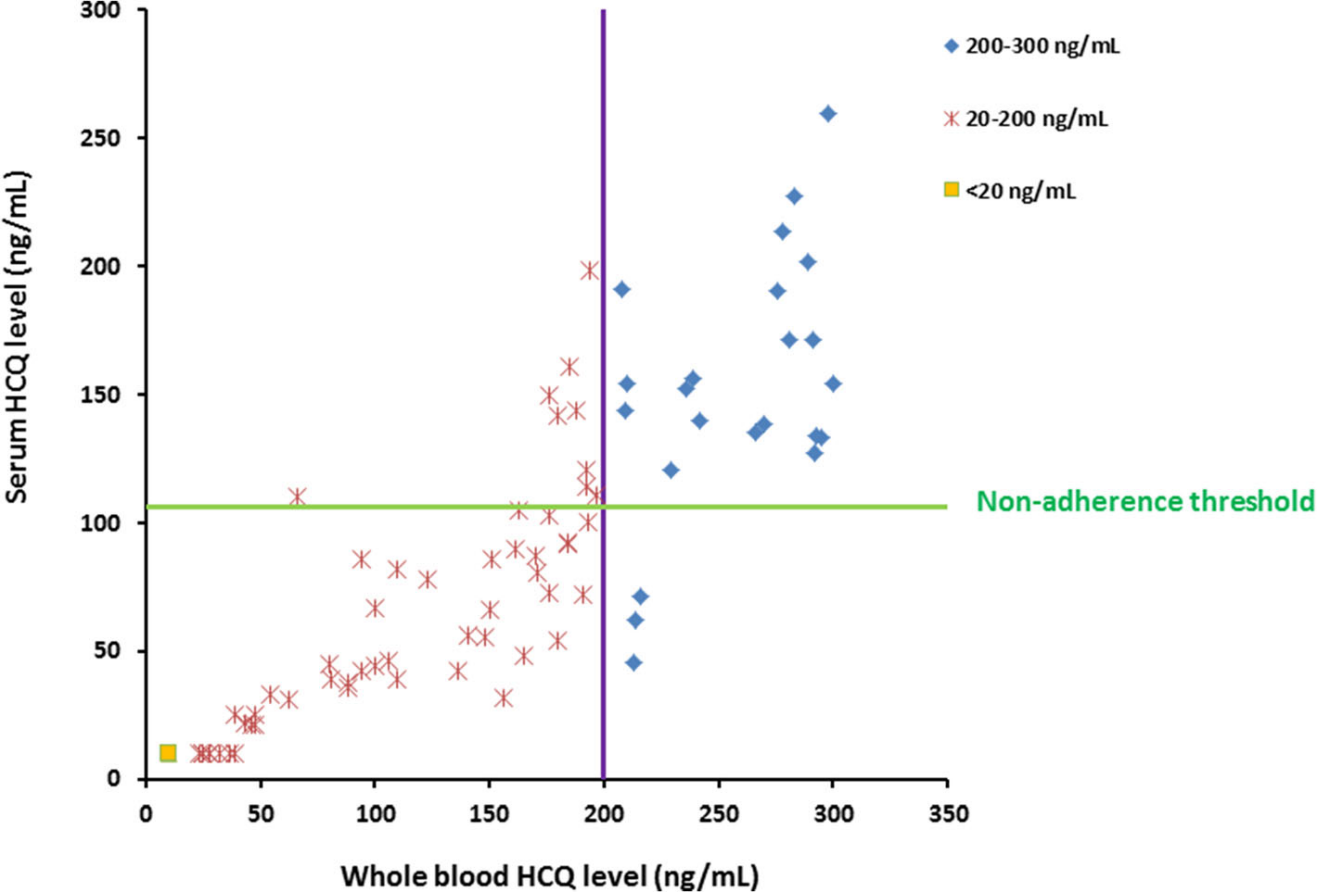
Relation between serum and whole-blood level of hydroxychloroquine (HCQ) in SLE patients with whole-blood levels < 300 ng/mL. The green and violet lines represent the HCQ level cut-off for non-adherence in serum (106 ng/mL) and whole blood (200 ng/mL), respectively. The orange square represents 14 patients who had both serum and whole-blood HCQ levels below the lower limit of quantification (20 ng/mL). Red crosses represent severe non-adherent patients with whole-blood HCQ levels between 20 and 200 ng/mL. Blue crosses represent patients with whole-blood HCQ levels between 200 and 300 ng/mL

With a serum HCQ cut-off of 106 ng/mL, 59 of the 68 patients with whole-blood HCQ levels below 200 ng/mL (87%) would also have been considered non-adherent according to their serum levels (Fig. 4). Of the 25 patients with whole-blood HCQ levels between 200 and 300 ng/mL, only 3 patients (12%) had a serum HCQ level below 106 ng/mL. These results yield a sensitivity of 0.87 (95% CI 0.76–0.94) and a specificity of 0.89 (95% CI 0.72–0.98). The positive and negative predictive values of serum HCQ < 106 ng/mL for detecting non-adherence defined by whole-blood HCQ < 200 ng/mL were 0.95 (95% CI 0.87–0.99) and 0.74 (95% CI 0.56–0.87), respectively. Finally, no patient with a whole-blood HCQ level > 300 ng/mL (*n* = 511) had a serum HCQ level < 106 ng/mL.

Among the 68 patients with whole-blood HCQ levels < 200 ng/mL, 37 had levels < 100 ng/mL, an alternative cut-off for severe non-adherence. With the corresponding serum HCQ cut-off of 53 ng/mL, this alternative definition would have considered 35 of these 37 patients (95%) non-adherent by their serum level. Of the 31 patients with whole-blood HCQ levels between 100 and 200 ng/mL, only 3 (10%) had a serum HCQ level < 53 ng/mL, for a sensitivity of 0.95 (95% CI 0.82–0.99) and a specificity of 0.90 (95% CI 0.74–0.98). The positive and negative predictive values of a serum HCQ level < 53 ng/mL for detecting the alternative definition of non-adherence were 0.92 (95% CI 0.79–0.98) and 0.93 (95% CI 0.78–0.99), respectively.

Finally, all serum HCQ levels of the 14 patients with whole-blood HCQ below the detectable levels (20 ng/mL) were also below the detectable levels for serum HCQ (20 ng/mL).

### Relation between whole-blood HCQ level and weight descriptor

To have homogeneous data, we restricted our analysis to the 522 patients treated with 400 mg/day of HCQ (Fig. 1). An inverse relation was observed between whole-blood HCQ level and dose per kg of TBW (rho = − 0.214 [95% CI − 0.294 to − 0.131], *P* < 0.0001), LBM (rho = − 0.212 [95% CI − 0.293 to − 0.129], *P* < 0.0001), and IBW (rho = − 0.111 [95% CI − 0.195 to − 0.026], *P* = 0.011). In patients weighing more than 90 kg (*n* = 33, 6.3%), no statistical relation was observed with any weight descriptors: TBW (*P* = 0.18), LBM (*P* = 0.60), or IBW (*P* = 0.68).

## Discussion

A PK/PD relation for HCQ has been shown in both serum and whole blood from SLE patients [5–7, 10–12, 17, 18, 20] but the interest of each approach has never been assessed. This study shows, as detailed below, that monitoring whole-blood levels appears more suitable than using serum levels for assessing the PK/PD relation in daily clinical practice. It also shows that serum and whole-blood HCQ levels correlate strongly (and better than for DCQ) and that the mean ratio of serum/whole-blood levels for HCQ were 0.53 ± 0.15. Additionally, it proposes for the first time serum HCQ cut-off levels to assess severe non-adherence, based on data from a large cohort of patients.

Studies addressing the PK/PD relation found that higher whole-blood HCQ levels were associated with less SLE activity and fewer flares [5–12, 29]. Using serum levels, Mok et al. also reported that SLE patients with serum HCQ levels > 500 ng/mL tend to have lower mean disease activity scores and a lower incidence of disease flares [12]. In agreement with this result, our study shows that patients with SLEDAI scores < 4 had higher HCQ serum levels than other patients (*P* = 0.008). However, in the multivariate analysis, only whole-blood HCQ levels were independently associated with active SLE (*P* = 0.023), an indication that whole-blood HCQ levels are more informative than the serum level about the PK/PD relation. It has been suggested that whole-blood measurements might be more reproducible and stable than serum measurements [8, 29]. In general, serum levels are valuable when the drug is not sequestered in red blood cells. Given that HCQ diffuses into these cells [30], the handling of samples, for example centrifugation, could influence HCQ partitioning between red blood cells and serum and produce misleading serum HCQ levels and thus a false pharmacological interpretation. The duration and force of centrifugation are known to significantly influence the levels of HCQ and DCQ in serum [31]. Red blood cell partitioning is also sensitive to temperature, pH, and blood collection procedures [32]. In addition, autoimmune haemolytic anaemia, which can occur in SLE, would probably modify serum HCQ levels considerably. All of these elements point out the need to minimise analytical variation by rigorous standardisation of centrifugation when serum is used for drug monitoring. Here, we observed substantial interindividual variability in HCQ and DCQ levels in both serum and whole blood. The magnitude of this variability was quite similar between the two biological matrices, probably because of the rigorous standardisation of centrifugation requested for the clinical trial. In this context, our study might have underestimated the interindividual variability in serum HCQ levels in daily clinical practice.

Since PLUS study failed to demonstrate the benefit of adapting daily HCQ dose to its whole-blood levels [9], drug monitoring is mainly recommended today to assess non-adherence to HCQ treatment in SLE patients [14]. We previously reported that patients with very low whole-blood HCQ levels admitted severe non-adherence to the treatment, and we proposed a cut-off of 200 ng/mL that has proved to be effective in our daily practice since then. Others have chosen different cut-offs (500, 100, < 15 ng/mL, or undetectable levels) or have used serum levels [5, 11, 12, 14, 16–19]. As far as we know, the present study is the first to propose serum HCQ cut-off points corresponding to our cut-off of 200 ng/mL (or 100 ng/mL as an alternative definition) to identify non-adherent patients. The strength of our study is that the ratio of serum/whole-blood HCQ could be determined from the data of 573 patients. At a serum HCQ cut-off of 106 ng/mL, the sensitivity was 0.87 (95% CI 0.76–0.94) and the specificity 0.89 (95% CI 0.72–0.98). HCQ levels undetectable by one method were also undetectable by the other. Further research to validate the best cut-off point for clinical practice requires confirmation in a larger cohort of SLE patients.

In this study, the best correlation of HCQ levels with dose per kg was observed with weight measured as TBW and LBM. Among patients weighing more than 90 kg, neither the dose per kg of LBM (*P* = 0.60) nor that of TBW (*P* = 0.18) was statistically associated with whole-blood HCQ. Nonetheless, this result should be interpreted with caution given the low number of patients (*n* = 33). LBM is known to correlate better with the pharmacokinetics of hydrophilic drugs than TBW does, especially with their volume of distribution, while TBW is a better parameter for lipophilic drugs [22]. The lipophilicity of HCQ may explain in part why the relation between whole-blood HCQ level and dose per kg of LBM is no better than that of dose per kg of TBW. Finally, the relation between whole-blood HCQ and dose per kg of IBW was worse than that with dose per kg of TBW. A French multicentre prospective study in patients with cutaneous lupus erythematosus similarly did not observe a relation between whole-blood HCQ and dose per kg of IBW. Interestingly, it has been recently shown that TBW also correlates better with retinal toxicity than IBW, which suggests that TBW should be used to prevent the onset of this ocular toxicity [33]. Taken together, these results confirm that TBW is more appropriate than IBW for determining the HCQ dose to be prescribed in SLE patients.

Our study has some limitations. First, it was necessary to enrich our PLUS cohort to obtain patients with different levels of SLE activity (since patients with severe SLE were not included in the PLUS study) and to have patients with severe non-adherence. Second, whole-blood HCQ levels were measured in 2 different laboratories but we have previously reported that the methods used by both laboratories are comparable [28]. Third, the evaluation of sensitivity and specificity of HCQ cut-offs in serum was based on data from a small cohort of non-adherent patients (*n* = 68). We note that our estimates are conservative: we used only patients with whole-blood HCQ levels between 200 and 300 ng/mL to calculate the specificity; it would have been much higher had we used patients with higher levels, since none of them had serum levels lower than 106 ng/mL.

In conclusion, our data support the use of whole blood rather than serum as the matrix for drug monitoring of HCQ levels in SLE patients to assess the PK/PD relation. However, when whole blood is not available, our results support the use of serum HCQ to assess non-adherence with a cut-off of 106 ng/mL, corresponding to 200 ng/mL and undetectable levels by one method also undetectable by the other.

## Supplementary information

**Additional file 1.**

## Data Availability

The datasets used and/or analysed during the current study are available from the corresponding author on reasonable request.

## Abbreviations

HCQ: Hydroxychloroquine
SLE: Systemic lupus erythematosus
PK/PD: Pharmacokinetic/pharmacodynamic
BMI: Body mass index
TBW: Total body weight
IBW: Ideal body weight
LBM: Lean body mass
ACR: American College of Rheumatology
DCQ: Desethylchloroquine
