# Supplementary figures and images for: Hydroxychloroquine levels in patients with systemic lupus erythematosus: whole blood is preferable but serum levels also detect non-adherence
Source: Arthritis Res Ther. 2020 Sep 25;22:223. doi: 10.1186/s13075-020-02291-z (PMC7517694; doi:10.1186/s13075-020-02291-z)

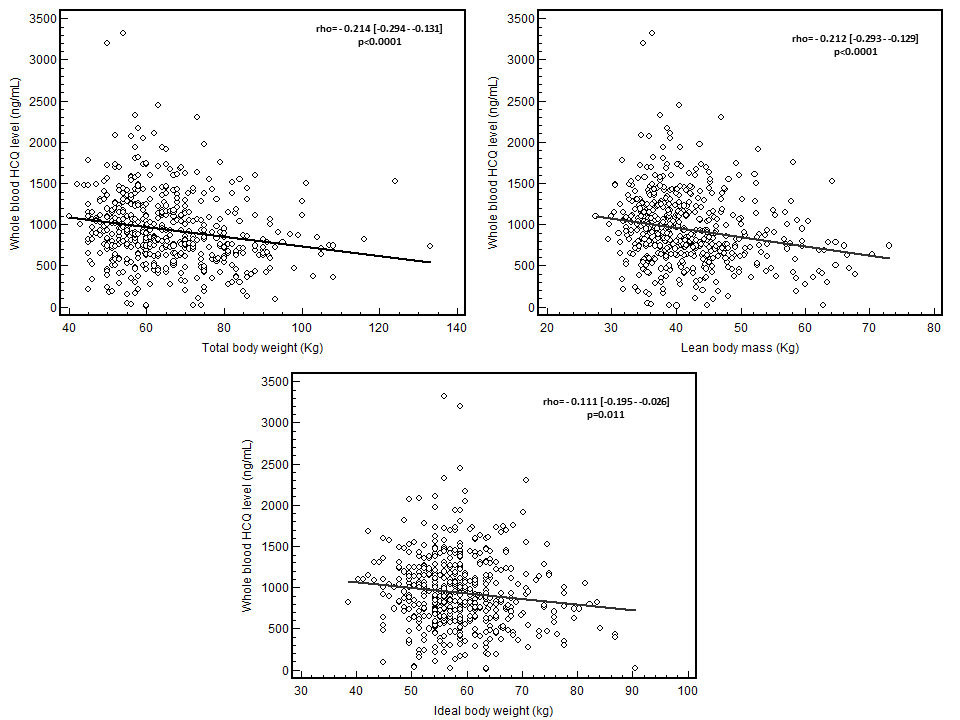

Supplement: Supplementary file 1 — Additional file 1. [file 13075_2020_2291_MOESM1_ESM.tif]
